# Downregulation of HLA-I by the molluscum contagiosum virus mc080 impacts NK-cell recognition and promotes CD8^+^ T-cell evasion

**DOI:** 10.1099/jgv.0.001417

**Published:** 2020-06-08

**Authors:** Hana Elasifer, Eddie C.Y. Wang, Virginie Prod’homme, James Davies, Simone Forbes, Richard J. Stanton, Mihil Patel, Ceri A. Fielding, Dawn Roberts, James A. Traherne, Nicole Gruber, Joachim J. Bugert, Rebecca J. Aicheler, Gavin W. G. Wilkinson

**Affiliations:** ^1^​ Division of Infection and Immunity, Cardiff University School of Medicine, Cardiff CF14 4XW, UK; ^2^​ Department of Pathology, University of Cambridge, Tennis Court Road, Cambridge CB2 1QP, UK; ^3^​ DKMS Life Science Lab, St. Petersburger Str. 2, 01069 Dresden, Germany; ^4^​ School of Sport and Health Sciences, Cardiff Metropolitan University, Cardiff CF5 2YB, UK; ^†^​ Present address: Centre Méditerranéen de Médecine Moléculaire, University of Nice Sophia, Antipolis, France; ^‡^​ Present address: Institut für Mikrobiologie der Bundeswehr, München, Germany

**Keywords:** MHC-I, *Molluscum contagiosum*, NK cell, T cell

## Abstract

Molluscum contagiosum virus (MCV) is a common cause of benign skin lesions in young children and currently the only endemic human poxvirus. Following the infection of primary keratinocytes in the epidermis, MCV induces the proliferation of infected cells and this results in the production of wart-like growths. Full productive infection is observed only after the infected cells differentiate. During this prolonged replication cycle the virus must avoid elimination by the host immune system. We therefore sought to investigate the function of the two major histocompatibility complex class-I-related genes encoded by the MCV genes mc033 and mc080. Following insertion into a replication-deficient adenovirus vector, codon-optimized versions of mc033 and mc080 were expressed as endoglycosidase-sensitive glycoproteins that localized primarily in the endoplasmic reticulum. MC080, but not MC033, downregulated cell-surface expression of endogenous classical human leucocyte antigen (HLA) class I and non-classical HLA-E by a transporter associated with antigen processing (TAP)-independent mechanism. MC080 exhibited a capacity to inhibit or activate NK cells in autologous assays in a donor-specific manner. MC080 consistently inhibited antigen-specific T cells being activated by peptide-pulsed targets. We therefore propose that MC080 acts to promote evasion of HLA-I-restricted cytotoxic T cells.

## Introduction

Molluscum contagiosum virus (MCV), a member of the genus *Molluscipoxvirus*, exclusively infects humans and is currently the only endemic human poxvirus [1]. MCV is a dermatotropic poxvirus that infects and induces hyperproliferation of epidermal cells, yet productive infection is realized only following keratinocyte differentiation and culminates in the release of mature virions at the tip of skin papules [2, 3]. While the MCV replication cycle is distinct from that of other characterized poxviruses, the transformation of keratinocytes has parallels with that of the papillomaviruses. Highly contagious, self-limiting MCV infections are primarily observed in children and can persist for months to years. The generally benign nature of skin lesions combined with their remarkable capacity to persist implies that MCV is extremely well adapted to its host. Spontaneous regression follows the induction of an inflammatory response that is characterized by the recruitment of natural killer (NK) cells, T cells and dendritic cells to skin lesions [4]. Serious persistent MCV infections with larger and more extensive lesions are indicative of an underlying immunodeficiency and are a recognized complication in severely immunocompromised human immunodeficiency virus - Acquired Immune Deficiency Syndrome patients [5].

The ~190 kb linear double-stranded DNA MCV genome is predicted to encode some 182 canonical protein-coding genes, including 105 that have homologues in Orthopoxviruses and 36 that share homology with cellular genes [6, 7]. Poxviruses apportion much of their coding capacity to provide for an arsenal of intrinsic, innate and adaptive immune evasion functions [8]. Although studies of MCV pathogenesis have been frustrated by its host restriction and the lack of a system to propagate the virus *in vitro*, the examination of MCV genes in isolation has revealed that MC054 sequesters interleukin-18, MC148 is a chemokine antagonist capable of suppressing chemotaxis and MC159 inhibits Tumour Necrosis Factor-induced NF-κB activation [9, 10].

Cytotoxic T lymphocytes (CTL) and NK cells have both been implicated in the effective control of poxvirus infections [11]. However, viruses have evolved a wide range of mechanisms to evade both CD8^+^ T cells and NK cells. Cowpox actively downregulates expression of the major histocompatibility complex 1 (MHC-I) proteins from the cell surface through a combination of the CPXV203 protein retaining MHC-I in the endoplasmic reticulum (ER) and CPXV12 preventing peptide loading [12–15]. In vaccinia virus (VACV) the host-cell shut-off function suppresses expression of both endogenous classical MHC-I and the non-classical human leukocyte antigen class-I antigen alpha chain E (HLA-E). In addition to their primary function of presenting peptide antigens to CD8^+^ T cells, MHC-I provide the chief ligands recognized by NK-cell inhibitory receptors; in main these include leukocyte immunoglobulin-like receptor 1 (LILRB1/CD85J/ILT2) and killer-cell immunoglobulin-like receptors (KIRs). Consequently, the suppression of endogenous MHC-I expression has the potential to render cells more vulnerable to NK-cell attack. HLA-E binds a restricted set of peptides normally derived in a TAP-dependent manner from the leader sequences of classical HLA-I molecules. HLA-E is a ligand for the NK-cell inhibitory receptor CD94/natural killer group 2 (NKG2)A. Loss of HLA-E from the surface renders VACV-infected cells more sensitive to CD94/NKG2A^+^ NK cells [16]. Moreover, the VACV haemagglutinin acts directly as a ligand for NK-cell activating receptors NKp30 and NKp46, yet the interaction with NKp30 is associated with NK-cell suppression [17].

Two human cytomegalovirus (HCMV) MHC-I homologues (UL18 and UL142) are well established NK-cell evasion functions, with gpUL18 binding LIR1 as a MHC-I mimic [18, 19]. Interestingly, cowpox and monkeypox viruses also encode a secreted MHC class-I-like protein (OMCP) that binds the NK-cell-activating receptor NKG2D with high affinity, allowing OMCP to act as an efficient antagonist of NKG2D [20, 21]. The MCV genome has also been shown to encode two ORFs with sequence similarity to MHC-I: mc033 and mc080 [6]. We were interested in exploring the possibility that these MCV MHC-I-related genes could play a role in evasion of NK cells. MC033 was originally identified as having greatest sequence similarity to Xenopus MHC-I [7]. The protein database has expanded over time and currently MC033 exhibits the closest similarity to a zinc-alpha-2-glycoprotein-like sequence present in Vombatus ursinus (common wombat). Although the Zinc-alpha-2-glycoprotein (ZAG) itself has a similar structure to MHC-I, it is a secreted protein known to stimulate lipid metabolism [22]. The sequence alignment between MC033 and MHC-like proteins in the database is restricted to ~115 amino acids near the C-terminus of MC033. In contrast, the similarity between MC080 and mammalian MHC-I heavy chains extends through the α1, α2 and α3 domains. When MC080 was expressed using a replication-competent vaccinia virus vector the protein was observed to accumulate in the ER and form a complex with ß−2 microglobulin, however its sequence was not thought to be compatible with peptide binding [23]. In a study undertaken in parallel with the one reported here, Harvey and co-workers recently reported that MC080 interacts with tapasin to impair cell-surface expression of MHC-I antigen [24].

In order to investigate whether the MCV MHC-I-like genes modulated NK-cell function, we expressed codon-optimized versions of MC033 and MC080 by using a replication-deficient adenovirus (RAd) vector. Ectopic expression of MCO33 had no overt impact on NK- or T-cell function, while MC080 was observed to both modify recognition by NK cells and to promote CD8^+^ T-cell evasion. Detailed investigation revealed MC080 downregulated cell-surface expression of both HLA-I and HLA-E.

## Results

### Characterizing MC033 and MC080 expression

**Fig. 1.**
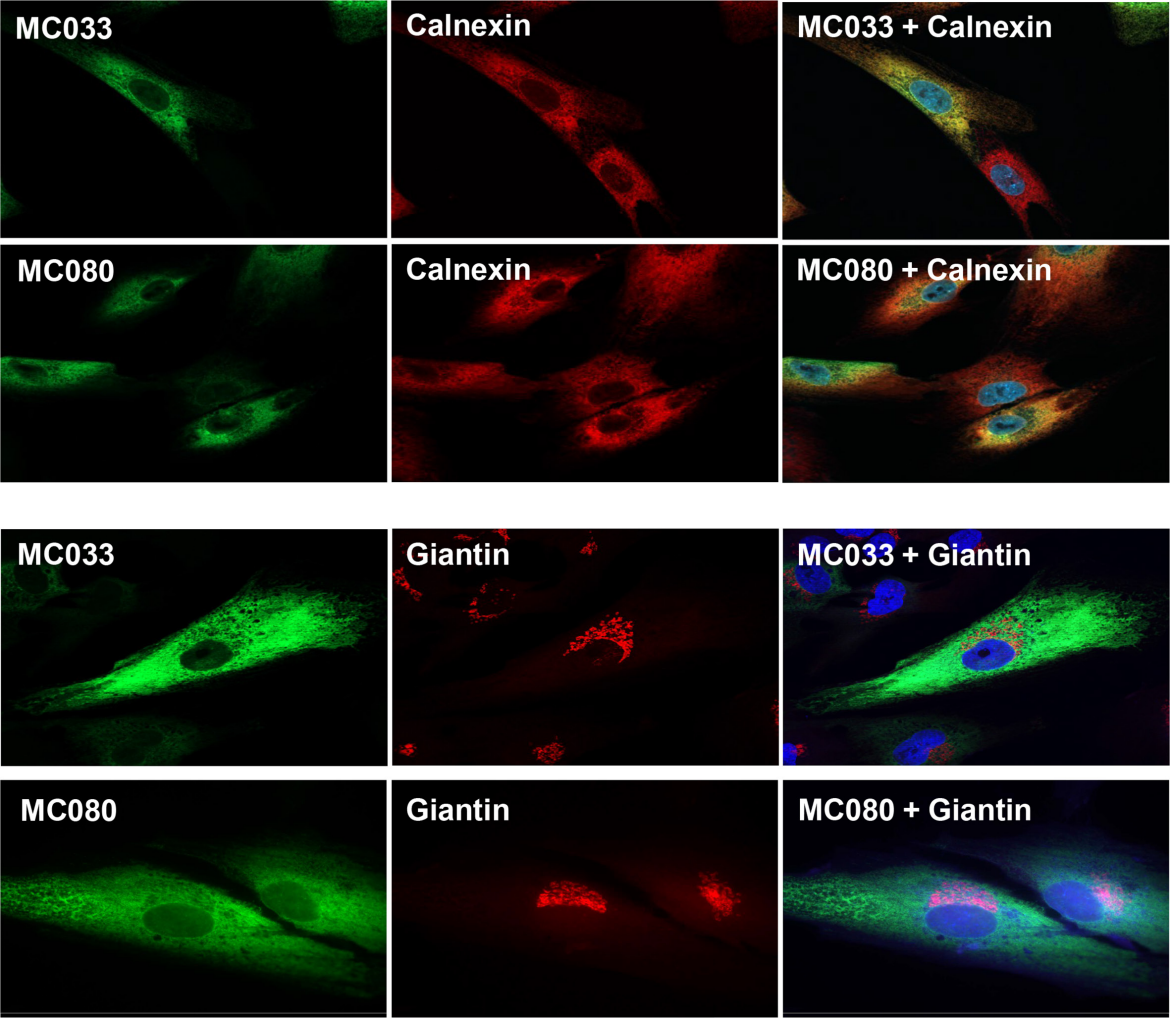
MC033 and MC080 localize to the ER. Immunofluorescence was performed on HF-CAR fibroblasts infected with RAd-mc033 or RAd-mc080 for 48 h (m.o.i.=5 p.f.u./cell). Expression of MC033 and MC088 was detected using an antibody to the C-terminal V5 epitope tag. Antibodies to calnexin and Giantin were used as ER and *cis*-Golgi apparatus markers (red) whilst DAPI provides a nuclear stain (blue) on the merged images. Results were consistent across two experiments.

The native forms of the mc033 and mc080 genes were synthesized in extremely low amounts and induced significant cytotoxicity when produced using mammalian expression vectors [25]. It is possible these issues may be related to the high GC content (63%) of MCV DNA. Codon-optimized versions of mc033 and mc080 genes were therefore synthesized and inserted into a RAd to generate RAd-mc033 and RAd-mc080. A C-terminal V5 epitope tag was included in each construct to track expression. The codon-optimized versions expressed efficiently and with minimal cytotoxicity following infection with the RAd recombinants. MC033 and MC080 were observed to co-localize with an ER-marker (calnexin), but to be relatively distinct from the Golgi apparatus marker (giantin), thus both MCV proteins traffic to the ER when expressed in isolation (Fig. 1).

**Fig. 2.**
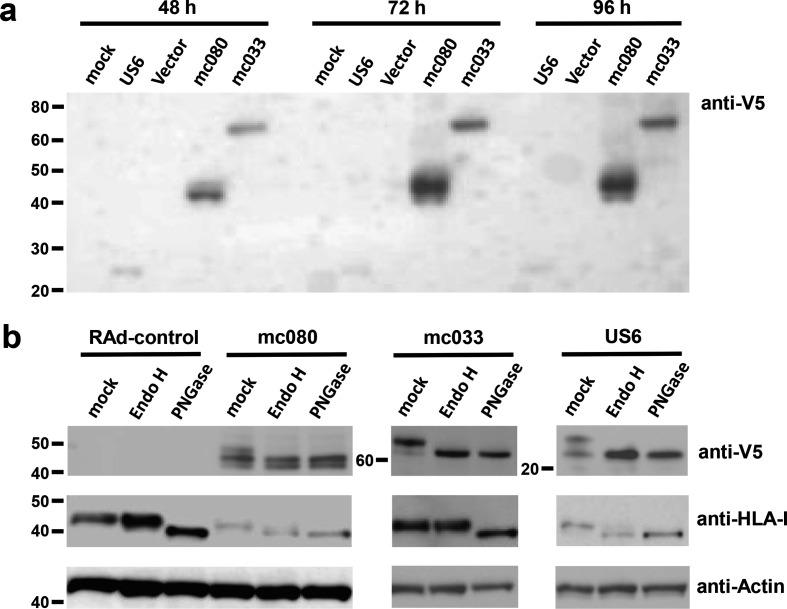
MC033 and MC080 protein expression. Samples prepared from RAd-mc033, RAd-mc080, RAd-US6 and RAd-control infected HF-CAR (m.o.i.=5) were analysed by Western blot. Expression of the viral proteins was detected by means of a C-terminal V5 epitope tag. (a) The time course illustrates stronger expression of MC033 and MC080 from 48 to 72 h p.i. than the positive control HCMV gpUS6. (b) Cell extracts from 72 h p.i. were treated with EndoH or PNGase. The expression of endogenous HLA-I expression was assessed using the HC10 antibody with actin detection serving as a loading control. Results were consistent across two experiments.

When RAd-mc033 and RAd-mc080 infected cells were analysed in Western blots, MC033 and MC080 were both expressed at peak levels by 72 h p.i. and at higher levels than a V5 epitope-tagged HCMV gpUS6 control (Fig. 2a). US6 encodes an ER resident glycoprotein that suppresses cell-surface HLA-I antigen presentation by inhibiting TAP [26]. The sensitivity of HCMV gpUS6 to endoglycosidase H (EndoH) is consistent with its retention in the ER (Fig. 2b). MC033 was produced as a 62 kDa glycoprotein and was susceptible to both EndoH and Peptide:N-glycosidase F (PNGase) digestion (Fig. 2b). Three closely migrating forms of MC080 were discernible with molecular masses calculated at 40, 42 and 46 kDa. The 46 kDa form must be glycosylated as it was sensitive to both EndoH and PNGase, whereas the 40 kDa species may be an immature unglycosylated precursor as it was unaffected by either glycosidase and co-migrated close to deglycosylated MC080. The 42 kDa form detected after PNGase treatment must also be deglycosylated and may be a product of differential cleavage at the N-terminus or some other form of post-translational processing. The sensitivity of MCV MC033 and MC080 glycoforms to EndoH was compatible with the immunofluorescence data that showed both proteins localized predominantly to the ER.

### Impact of MC033 and MC080 on NK cells

**Fig. 3.**
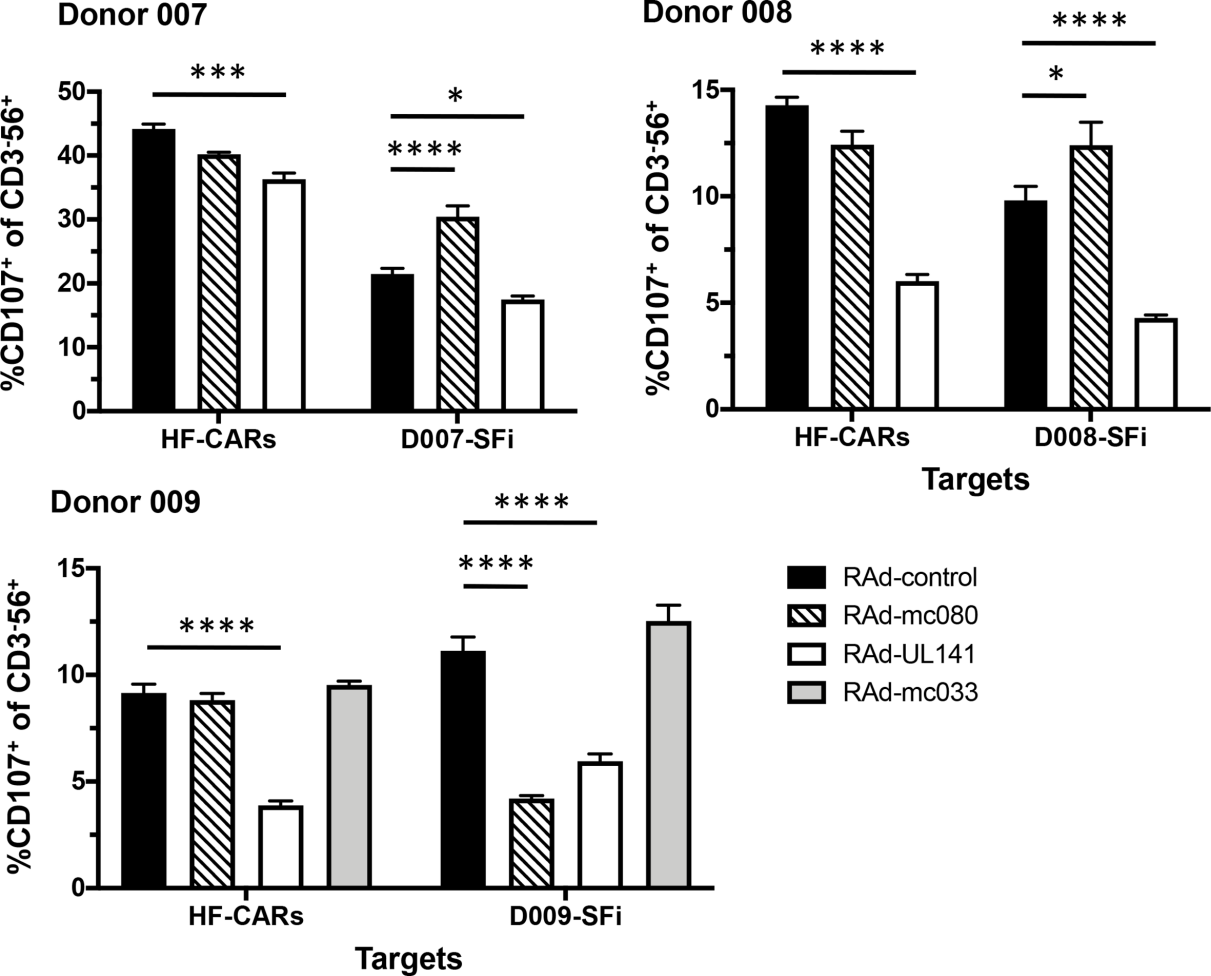
MC080 modulates NK-cell activity. The effect of MC080 and MC033 on NK-cell degranulation was assessed by measuring the proportion of CD107a^+^ cells within the CD3^-^CD56^+^ population. HF-CARs and autologous skin fibroblasts cells (D007-SFi, D008-SFi or D009-SFi) were infected at a m.o.i. of 5 or 500, respectively, with RAd-mc080, RAd-mc033, RAd-UL141 or the vector (RAd-control) for 72 h. The higher m.o.i. is required to provide for comparable infection and transgene expression in the primary skin fibroblasts that exhibit minimal Ad receptor (hCAR) expression. Infected cells were incubated with donor PBMC with PBMC:target ratio of 10 : 1. NK-cell degranulation was assessed by measuring %CD107a^+^ cells within a CD3^-^CD56^+^ gated population within the lymphocyte gate. Results (shown as mean+sem) were analysed by two-way ANOVA with Tukey’s multiple comparison test; *P<0.05, ***P<0.001, ****P<0.0001. Data from three of four experiments in three donors shown.

We sought to investigate whether the MCV MHC-I-like genes were also capable of modulating NK-cell function. Peripheral blood mononuclear cells (PBMC) isolated from healthy volunteer donors were stimulated with interferon-α and deployed in a NK degranulation assay. A standard human foetal foreskin fibroblast line (HF-CAR) served as allogeneic targets whereas fibroblasts cultured from donor skin biopsies permitted NK-cell function to be assessed in an autologous setting. The HCMV NK-cell evasion gene UL141 [27], included as a positive control, inhibited NK-cell activation in all experiments (Fig. 3). MC033 expression in HF-CAR had no obvious effect on NK-cell activation in donors 008 and 009, the elevated level observed in autologous skin fibroblasts with donor 009 did not reach statistical significance (Fig. 3) and further studies were unable to detect any effect of MC033 on NK-cell activation (not shown). The situation with MC080 was more complex. When HF-CARs were used as targets, MC080 had no significant impact on donors 007, 008 or 009 NK-cell function; the reduced level of NK-cell activation with donors 007 and 008 did not reach statistical significance (Fig. 3). In an autologous setting, however, MC080 was observed to be capable of both activating (donor 007 and 008) or inhibiting NK-cell degranulation (donor 009) (Fig. 3). Thus MC080 was capable of modulating NK-cell function, either suppressing or activating the response depending on properties inherent in the effector and target cells.

**Fig. 4.**
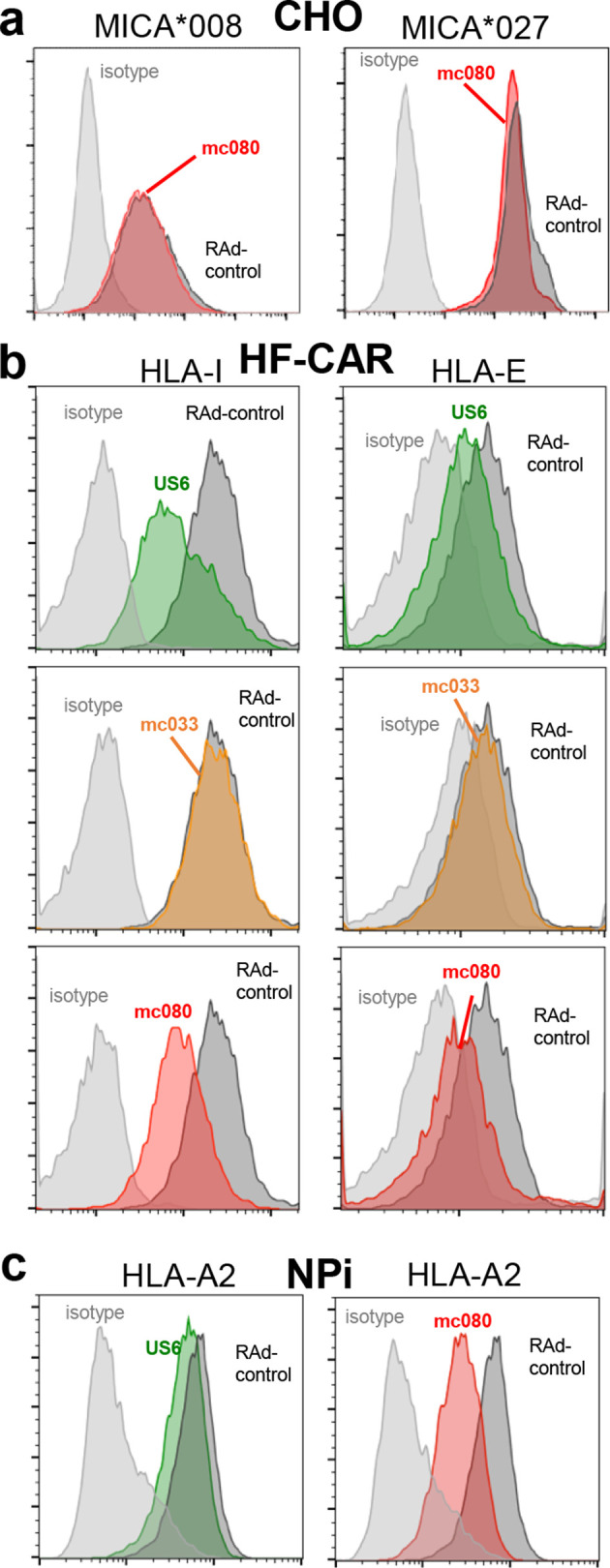
MC080 suppresses cell-surface expression of HLA-I and HLA-E. Flow cytometric analysis was performed to investigate the effect of MC080 on MICA, HLA-E and HLA-I. (a) CHO cells were co-infected with RAd-mc080 and an RAd vector encoding MICA, either MICA*027 or MICA*008 alleles, or the vector (RAd-control) for 72 h. Each virus was used at a m.o.i.=50 p.f.u./cell and cells stained with phycoerythrin (PE) conjugated anti-MICA or a PE conjugated isotype control antibody. (b) Cell-surface HLA-I and HLA-E were measured in HF-CAR cells infected with RAd-mc033, RAd-mc080, RAd-US6 or a vector control (m.o.i.=5 p.f.u./cell) for 72 h. (c) NPi cells, which have a defect in the TAP transporter, were infected with Rad-HLA-A2 together with RAd-control, RAd-US6 or RAd-mc080. Each virus was used at a m.o.i.=500 p.f.u./cell and cells stained with allophycocyanin (APC) conjugated anti-MHC-I or an APC-conjugated isotype control antibody. Results were consistent across four experiments.

HCMV UL142, a MHC-I homologue, has been reported to downregulate the full-length allelic forms of the NKG2D ligand MHC class I polypeptide-related sequence A (MICA) yet not the C-terminally truncated MICA*008 variant [28, 29]. We hypothesized that our results could be explained if MC080 exhibited a similar selective interaction with full-length and truncated MICA*008 alleles. HLA and NK-cell receptor genotypes for the donors and cells used in this study are detailed in Table S1 (available in the online version of this article). Remarkably, MICA genotyping revealed that donors 007, 008 and 009 were homozygous for the same truncated MICA*008 allele (specifically MICA*00801/04) whereas HF-CARs possess full-length MICA alleles (MICA*016 and MICA*027). Moreover, MC080 was not able to suppress cell-surface expression of either the truncated MICA*008 or full-length MICA*027 when expressed in CHO cells, although we cannot exclude the possibility that such an MC080 function could require a species-specific factor (Fig. 4a). The differential donor-specific effect of MC080 on NK-cell responses is thus unlikely to operate through MICA.

Endogenous HLA-I molecules provide the chief ligands for a range of NK-cell inhibitory receptors that include the family of KIRs, LIR1 and CD94/NKG2A. However, certain HLA-I molecules can also be recognized by activating KIRs. The balance of signals to NK cells through KIR/HLA-I interaction will naturally be different between donors. A comparison of the KIR genotypes revealed that D007 and D008 are homozygotic for the KIR A haplotype whereas D009 carries a KIR A and a KIR B (adding extra activating receptors) haplotype (Table S1). D007 and D008 possess an additional copy of the inhibitory KIR3DL1 allele, when compared to D009. D009 possesses three activating KIRs (3DS1, 2DS1 and 2DS5) and one inhibitory KIR (2DL5) that are not present in D007 or D008. On balance, the KIR repertoire of D009 is more activating than that of D007 or D008. We therefore speculate that the capacity of MC080 to either suppress or activate NK-cell recognition depends on properties bestowed by a combination of donor and target cells and could be brought about by modulating HLA-I expression.

### MC080 downregulates expression of HLA-I

HLA-I expression on human fibroblasts infected with RAd-mc080, RAd-mc033 and RAd-US6 was analysed by flow cytometry. MCV MC080 and HCMV gpUS6 reduced the levels of surface HLA-I surface expression, whereas the effect of expressing MC033 was comparable to the vector control (Fig. 4b). MC080 was also found to have a marked effect on total cell expression of endogenous HLA-I in Western-blot experiments (Fig. 2b). Not only were the levels of HLA-I heavy chain (HC) markedly reduced in cells expressing MC080, the HC was rendered susceptible to Endo-H digestion thus implying it was being retained in the ER. HCMV US6, an inhibitor of TAP [26], and MC080 both had a similar effect on HLA-I whereas MC033 had no obvious effect on cell-surface HLA-I expression or its susceptibility of HLA-I to Endo-H digestion.

HLA-E is a non-classical HLA-I molecule that modulates NK-cell function through its interactions with the inhibitory receptor CD94/NKG2A and the activating receptor CD94/NKG2C. Interestingly MC080 and gpUS6, but not MC033, also downregulated cell-surface expression HLA-E (Fig. 4b). HLA-E binds a highly restricted set of peptides that are normally derived from the signal peptide of classical HLA-I molecules. Cell-surface expression of HLA-E is TAP-dependent and thus inhibited by US6 [30]. It was possible that MC080 could also be suppressing HLA-I and HLA-E cell-surface expression by targeting TAP. A TAP2 deficiency in the NPi fibroblast cell line is responsible for low level surface HLA-I and HLA-E expression [31]. However, the HLA-A*02 allelic group is remarkable in binding hydrophobic peptides that enter the ER independently of TAP [32]. When an adenovirus encoding HLA-A2 was introduced in to NPi cells, the cell-surface HLA-A2 expression realized in the TAP-deficient cells proved to be resistant to gpUS6 yet sensitive to MC080 (Fig. 4c). MC080 is therefore able to suppress cell-surface HLA-A2 in human cells lacking TAP2.

### MC080 expression in target cells inhibits T-cell activation

**Fig. 5.**
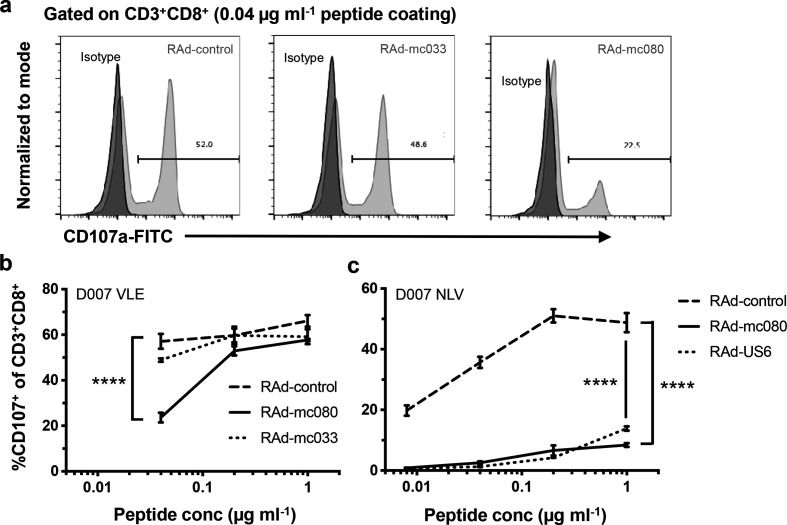
MC080 suppresses T-cell activation. Donor 007 (D007)-SFi were infected with RAds encoding mc080 (RAd-mc080), mc033 (RAd-mc033), HCMV US6 (RAdUS6) or the same vector without a transgene (RAd-control). Cells were harvested 72 h p.i. and coated with various concentrations of the HCMV IE1 VLE peptide (0.04 µg ml^−1^, 0.2 µg ml^−1^, 1.0 µg ml^−1^) to act as targets in a CD107a degranulation assay with a D007 HCMV VLE-specific CTL line as effectors. (a) Flow cytometric histograms showing CD107a degranulaton at 0.04ug ml^-1^ peptide coating. (b) Graph depicting CD107a degranulation over a range of peptide concentrations. One of two experiments shown. (c) D007-SFi cells were infected with the RAds indicated (72 h) then coated with HCMV pp65 NLV peptide (0.008 µg ml^−1^, 0.04 µg ml^−1^, 0.2 µg ml^−1^, 1.0 µg ml^−1^). A CD107a degranulation assay was performed using a D007 HCMV NLV-specific T-cell line as effectors. HCMV US6, a TAP inhibitor, was included as an HLA-I downregulating control. Results (shown as mean±sd of quadruplicates) were analysed by two-way ANOVA with Tukey’s multiple comparison test, ****P<0.0001.

To study the effect of MC080 on CD8^+^ T cells, CD107a assays were performed to measure effector cell degranulation when exposed to different targets. Since a tractable MCV system was not available, two HCMV HLA-A*0201-restricted T-cell lines were used, one specific for the IE1 epitope VLEETSVML (VLE) and a second for the pp65 epitope NLVPMVATV (NLV). The T-cell lines were tested in an autologous setting by using human epidermal fibroblasts cultured from a skin biopsy. MC033 exerted no significant effect on T-cell function (Fig. 5). While MC080 induced clear protection against T-cell degranulation induced by the VLE peptide, this protection could be overcome by increasing the peptide concentration (Fig. 5b). In contrast, MC080-induced protection against T-cell activation by the NLV peptide operated across a wide range of peptide concentrations (Fig. 5). The capacity of increasing peptide to rescue activation at the concentrations tested in only one of the T-cell lines may reflect differences in the affinity of the TCRs for their cognate peptide/HLA-I complex. MC080 expression thereby bestowed protection against T-cell activation in functional assays.

## Discussion

In the absence of an *in vitro* system for propagating MCV, the two MCV MHC-I-like genes were expressed using a RAd vector. When cloned as their native gene sequences, both genes expressed extremely inefficiently and induced overt cytotoxicity. It is possible that the high GC content of MCV DNA may have caused issues in the mammalian vectors as the issue was overcome when their codon usage was optimized. Both MC033 and MC080 were synthesized as EndoH-sensitive glycoproteins that localized to the ER. While MC033 aligns with a MHC-I-like protein, the sequence similarity is low and restricted. MC033 lacks homology through the MHC-I α1 and α2 domains responsible for peptide binding and required for many MHC-I receptor interactions. The inability of MC033 to impact NK-cell function in our assays is consistent with it not acting as a simple MHC-I mimic.

MC080 is an EndoH sensitive, ER-associated glycoprotein that downregulates expression of endogenous HLA-I and HLA-E. MC080 was able to suppress cell-surface expression of classical HLA-I molecules and HLA-E in both TAP-positive and TAP-negative human cells. Moreover, post-translation maturation of HLA-I in the Golgi apparatus was suppressed by MC080. These findings implied that MC080 may be acting in the ER by direct sequestration or through competition for a factor required for HLA-I maturation. Further insight comes from a recent study that identifies an interaction between MC080 and tapasin as being critical in suppressing MHC-I maturation in murine cells [24]. Our observation that MC080 suppressed surface expression of HLA-A2.1 in TAP2-deficient human fibroblasts (Fig. 4) would be consistent with this model if tapasin retained a substantial role in loading TAP-independent peptides on to HLA-A2 in TAP-negative fibroblasts. Alternatively, MC080 may possess an additional mechanism for promoting MHC-I retention in human cells.

The targeting of HLA-E appears counterintuitive since it is recognized by the NK-cell inhibitory receptor CD94/NKG2A. Indeed, the downregulation of HLA-E by VACV proved sufficient to render infected cells sensitive to NKG2A^+^ NK cells [16]. Moreover, the upregulation of HLA-E cell-surface expression mediated by HCMV UL40 bestows protection against NKG2A^+^ NK cells [31, 33]. However, HLA-E can also be recognized by the paired NK activating receptor CD94/NKG2C and by HLA-E-specific T cells; expansions of CD94/NKG2C^+^ NK cells are commonly associated with HCMV infection [34–37]. A change in the target cell from allogeneic HF-CARs to autologous skin fibroblasts had a dramatic effect on NK-cell recognition (Fig. 3). Since HLA-E exhibits only limited sequence variation, it seemed unlikely the differential effects produced by MC080 expression on NK cells can be attributed to regulation of HLA-E.

In downregulating endogenous HLA-I, MC080 removes the natural ligand for multiple inhibitory or activating KIRs. The downregulation of a ligand for an inhibitory KIR would be expected to stimulate NK-cell function, as observed in an autologous setting for donor 007 and donor 008, whilst removal of the ligand for an activating ligand could result in NK-cell suppression, as observed in an autologous setting for donor 009. Donor 009 possessed a high number of activating KIRs (Table S1). The result illustrates the value in performing human NK assays in an autologous setting and revealed, in at least one individual, MHC-I downregulation by MC080 could be associated with reduced NK-cell activation. As many viruses suppress endogenous MHC-I, it will be interesting to study further exactly how KIR usage impacts sensitivity to virus infection.

MCV is remarkable in being able to sustain a persistent infection for many months with efficient virus production from overt lesions in immunocompetent individuals. MC033 and MC080 currently exhibit the closest sequence similarity with the common wombat ZAG and wild boar (*Sus scrofa*) MHC-I, respectively. The fact that MC080 and MC033 do not align closely with a human MHC-I implies there has been an evolutionarily complex route from host capture to the MCV genome. The ancient nature of their gene capture is further supported by the identification of homologues to both mc033 and mc080 in the recently characterized equine molluscum contagiosum-like virus (Ehmann *et al*., in press) [38]. While the function of MC033 has still to be defined, the modulation of endogenous HLA-I expression by MC080 is associated both with the evasion of MHC-I restricted cytotoxic T cells and modulating NK-cell activation.

## Methods

### Ethics statement

Written informed consent was obtained from healthy adult volunteers who kindly provided blood and dermal fibroblasts using procedures approved by the Cardiff University School of Medicine Research Ethics Committee Ref. no: 10/20 and 16/52.

### Cell lines

HF-CAR cells are human foetal foreskin fibroblasts transduced with retroviral vectors encoding the Coxsackie-adenovirus receptor and human telomerase [39]; dermal fibroblasts were cultured directly from skin biopsies. NPi cells are human fibroblasts with a genetic defect in TAP2, kindly provided by Professor Vincenzo Cerundolo (University of Oxford, UK). Chinese hamster ovary (CHO) cells were a kind gift of Dr Alan Parker (Cardiff University, UK). RAd vectors were cultured in 293-TREx cells [40]. All cells were grown in Dulbecco’s modified Eagle’s medium (DMEM) 10 % foetal calf serum (FCS) supplemented with penicillin/streptomycin (Invitrogen, Paisley, UK) except for HCMV-specific T-cell lines, which were expanded by co-culture with irradiated peptide-coated autologous fibroblasts in RPMI medium (10 % FCS, 2 % human AB serum, and 10 IU ml^−1^ interleukin-2). Primary NK cells were cultured as described previously [18, 36].

### Viruses

Codon-optimized versions of mc033 and mc080 (Figs S1 and S2) were produced by gene synthesis (Eurofins MWG Operon, Ebersberg, Germany) and amplified by PCR using primers containing arms of homology to permit direct insertion into the AdZ BAC vector (pAL1141) by DNA recombineering [40]. RAd-US6 and RAd-UL141 contain HCMV US6 and UL141 respectively and have been described previously [41, 42]. The MICA*027 and MICA*008 genes cloned into the RAd vector were obtained from Professor Dan Davis (University of Manchester, UK) and Professor Paul Lehner (University of Cambridge, UK), respectively. The titres of Ad vectors and recombinants were determined by plaque assay on 293-TREx cells. The infection efficiency of HF-CARs and 293-TREx cells with RAd vectors is identical. However, primary skin fibroblasts have minimal hCAR expression, and needed to be infected with 500 p.f.u. (evaluated in permissive 293 TREx cells) per cell to achieve efficient transgene delivery and expression in all cells.

### Western blot

EndoH and PNGase were used according to the manufacturer’s instructions (New England Biolabs, Hitchin, UK). Murine monoclonal antibodies were used to detect human HLA-I heavy chain (HC10 hybridoma kindly provided by Professor Hidde Ploegh, Boston Children’s Hospital, USA) and the V5-tag (MCA1360, cloneSV5-Pk1, BioRad, Watford, UK) while rabbit polyclonal antibody specific for actin (A2066, Sigma-Aldritch, Gillingham, UK) was used to check sample loading. Western blots were performed as described previously [27].

### Flow cytometry on adherent cells

Fibroblasts or CHO cells were removed from monolayers using trypsin and resuspended in PBS. Anti-MHC-I-allophycocyanin (APC) (clone W6/32; Biolegend, London, UK) or anti-HLA-E-phycoerythrin (PE) (clone 3D12; Biolegend) were incubated with the cells (30 min at 4 °C). Mouse IgG2a-APC (clone MOPC-173; Biolegend) and IgG1a-PE (clone MOPC-21; BD Biosciences, Oxford, UK) were used as isotype controls for HLA-I and HLA-E, respectively. Cells were then washed and fixed in 2 % paraformaldehyde (PFA) for 10 min and analysed using an Accuri C6 flow cytometer (BD Biosciences, Oxford, UK).

### Immunofluorescence staining and analysis

Immunofluorescence was performed as described previously [25, 43]. Cells were stained with rabbit anti-V5-tag (ab 9116, Abcam, Cambridge, UK), mouse anti-calnexin (clone C8.B6, Merck Millipore, Watford, UK), polyclonal goat anti-mouse Alexa Fluor 594 (Invitrogen) and goat anti-rabbit Alexa Fluor 488 (Invitrogen), anti-Giantin polyclonal sera (ab24586, Abcam), 0.5 µg ml^−1^ 4,6-diamidino-2-phenylindole dihydrochloride (DAPI) (Thermo Fisher), polyclonal goat anti-rabbit Alexa Fluor 594 (Invitrogen), and polyclonal goat anti-mouse-Alexa Fluor 488 (Invitrogen) as indicated and visualized using a Zeiss Axiomat fluorescence microscope (Carl Zeiss Microscopy, Germany).

### NK degranulation assays

NK degranulation assays were performed as described previously [18, 41]. Briefly, 5×10^5^ PBMC stimulated overnight with interferon-α (1000 IU ml^−1^) were incubated in triplicate with 5×10^4^ fibroblast targets and fluorescein isothiocyanate (FITC)-conjugated anti-CD107a mAb (clone H4A3, BD Biosciences) or mIgG1κ-FITC isotype isotype control (BD Biosciences). GolgiStop (BD Biosciences) was added after 1 h of incubation and the cells were incubated for a further 4 h. Cultures were washed with cold phosphate buffered saline (PBS), and stained with anti-CD3 PE-cyanine7 (clone 17A2, Biolegend) and anti-CD56 PE (clone N901, Beckman Coulter, High Wycombe, UK) mAbs for 30 min at 4 °C. Cells were washed twice in cold PBS, fixed in 2 % PFA and analysed using an Accuri C6 flow cytometer (BD Biosciences).

### T-cell assays

Autologous human fibroblast targets were coated with the indicated concentrations of the HLA-A2 restricted IE1 peptide VLEETSVML (VLE) or pp65 peptide NLVPMVATV (NLV) in 1 ml RPMI 10 % FCS for 1 h at 37 °C and washed twice with RPMI 10 % FCS to remove excess peptide. Peptide specific CD8^+^ T cells (10^5^) were incubated for 5 h with targets (10^4^ fibroblasts) in quadruplicate in the presence of FITC-conjugated anti-CD107a mAb (BD Biosciences). GolgiStop was added after 1 h of incubation. Cultures were washed with cold PBS, and stained with anti-CD3-peridinin-chlorophyll-protein-cyanine5.5 (clone SK7; BD Biosciences) and anti-CD8-APC (Biolegend) mAbs for 30 min at 4 °C. mIgG1κ-FITC (BD Biosciences) was used as an isotype control. Cells were washed twice in cold PBS, fixed in 2 % PFA and analysed using an Accuri C6 flow cytometer (BD Biosciences) [36].

### KIR genotyping

KIR copy number was determined using a multiplexed real-time PCR method as described previously [44]. KIR alleles were genotyped by Next Generation Sequencing based on amplification of exons 3, 4, 5, 7, 8 and 9 of all KIR genes as described previously [45].

## Supplementary Data

Supplementary material 1
