# Supplementary material for: Downregulation of HLA-I by the molluscum contagiosum virus mc080 impacts NK-cell recognition and promotes CD8+ T-cell evasion
Source: J Gen Virol. 2020 Jun 8;101(8):863–72. doi: 10.1099/jgv.0.001417 (PMC7641395; doi:10.1099/jgv.0.001417)
Supplement: Supplementary material 1 [file jgv-101-863-s001.pdf]

Supplemental Table 1 – Genotyping of donors and target cells

Genes	Donor		
	D007	D008	D009
HLA-A	A2, A24	A2, A24	A1, A24
HLA-B	B44, x	B60, x	B8, B44
MICA	00801/04, x	00801/04, x	00801/04, x
NKG2C*	-	+	+
KIR3DL3†	2	2	2
KIR2DS2†	-	-	-
KIR2DL2†	-	-	-
KIR2DL3†	2	2	2
KIR2DP1†	2	2	2
KIR2DL1†	2	2	2
KIR3DP1†	2	2	2
KIR2DL4†	2	2	2
KIR3DL1†	2	2	1
KIR3DS1†	-	-	1
KIR2DL5†	-	-	1
KIR2DS3†	-	-	-
KIR2DS5†	-	-	1
KIR2DS1†	-	-	1
KIR2DS4†	2	2	1
KIR3DL2†	2	2	2

x represents homozygous for allele

* genotype determined through staining for NKG2C on lymphocytes; nd = not done

† copy number of indicated KIR

Supplementary Fig 1: Codon optimized mc033Lwith C-terminal V5epitope. A) DNA sequence of codon optimised DNA inserted in to RAd-mc033 and B) Translation product showing location of C-terminal tag.

A) DNA Sequence

ATG AGG CCC CAC GTG CTG ATC ACC CTG GCC ACT TGT GCC TTG
CGG GCT CTT GCC CAG GTG ATA GAC GAA CAC GAG CAC TCT GAA
CCA CCA GTT TCA ACA TGG CCC GAC ATG TCC TAC CTG GTC GCA
GAG ATG CGG TCT GAT AGC GTA CTG ATG CGG GGC ATG CTG GAT
GGC CAT GAG CAC GTG CGC TGT ACT TGC GTG CCA AAG TGC GCC
TGC TTG GAG CCC ACC CTT CCA CGA GCC GCT CTG GAA CAA GCC
CGC TCA CGG GTA CTG GAT GCC CAT GCT GGT CGT GTG CCC GGA
CTG AGA GCC CCT AGC AGG GCA GCA CAT CGC AGA GTC GTG CTC
ACT GCC GGG TGT CGC TTC ACT CAG GGC TTT CCG GAG CCC TTT
GAG GGC CTC TGG GTC GCC AGC GCT GAA CCA GGT GCT CAC GAC
GAG TTC TTT TGT GTC GGT GAG CAC TGT GAC GCA CAC CTG TCC
TCC ATC TTT TGC CAC GCA GCA TCC ACC ATG CCC CTG GCC AGA
GCC CCT CAT AGC CCT CCC ATG GTG ACG TTC AGT GCC CTG TCA
GCC GGC GAA AAC TAC CTC AGG CTG GTT TGC CGG GCG TCT GGA
GCC TAT CCT CCT GTT GAC ACT CTG ACC CTC GTG AGC CAG CCT
CAA CAG CCA GAA GAT GCC CCT TGC GAG ACA TAC GCC GGA ACA
AAC GCT GAC AGC ACC GGC CAC GTC GGA ATG GCT TGC GTC CGT
TCT GAC GCG CTC GCT GGG GCA GCG TGT GCA GTC CAG CAT AGA
GGC GTG ACA ACC AGC GCC CGG ATT GTG CTT GTG CCG GCA AAT
GAT GGG GCC AAA GTC GGA GCC TAT GCT GAT GTC GAT GCC GAT
TTC TAT GCC GAC GTT CCG CCT CTC CCC GAA CCC GAG TCC GAC
AGC TTG GCT GTA CAC GCA CTG TTC GTA GCC GGG AAC ACT GAG
CTG TAT GTT CAC GGG ACA GCG GCT GGC GTA CCA TCT GCC TCC
TGT AGG TGT GAC ACC AGG AGA TGC ACA TGC GTG CTG GCT CCC
GCC ACA TGG ACT GCC GGA GTG GTG CGA GAA CTG GCG AGA GCG
GCT GCT CAT GAC CTT CTG CTT GCT GTG CTG GAC GTT CAT GCC
TCA GGC CTC GCT CTC AAC CGA TCA TCC ATG CAA GTG TAT GCC
GAA TGT GGG CCT GCA GGG AGG AGA CTT CGG GTT CAC AAT ACC
GGT ACC CGG CGA CAG CGC GTG TGC GCA CGC GGA GCT TGC GAG
CCG GCT TAT CTG GTG GCA TGT GAG CTG TTG CGG ACG GAC ACC
CCA GCA CCT CGG CGA CCT CGC ATG AGT GTT CAG CAC AGA CGG
GAT AGT AGC GGG CAT TAC TAC GTG TGC TCT GCC TAC GGC TTT
TAC CCC AAG GAG ATT GTG CTG GAG ATG AGA GCA AAT CGC AGC
TGT GAT GAG CGC GCA CGC CTG TCT GGT TTC TGG TGC AGA CAC
GAT CCA CCC GCA CCT AAT GCC GAT GGC ACC TTC TTT GCG AGG
GTG TTC TGC AAA GCG CCA GAA AAC GCC CTT CTG ATG ACC TGT
GTT ACA CGT CAT GCC AGT CGC CCA CGG GCC CTG GCC GTA CCA
TGT CCC CGA CGA GCT CGT ACT CCT AGG GAA AGA TGG GCC GCA
CTG CTC ACG GTG TTG GCA CGC GTC CCC TGG AGT GCT GTG CTG
TTG GCG CTG GCC ATG GGA GCA GCA CCG TTG GCT TGC GCT AGG
CTC GTC CAT GCT AGG TCC ACA AGG ACG GCC CGG AGA GCT AGG
CGT GCT AGG AGA GCG GGT AAG CCA ATC CCT AAC CCG CTC CTA
GGT CTT GAT TCT ACG TGA

B) MC033 amino acid sequence

MRPHVLITLATCALRALAQVIDEHEHSEPPVSTWPDMSYLVAEMRSDSVLMR
GMLDGHEHVRCTCVPKCACLEPTLPRAALEQARSRVLDAHAGRVPGLRAPSRAAHR
RVVLTAGCRFTQGFPEPFEGLWVASAEPGAHDEFFCVGEHCDAHLSSIFCHAASTM
PLARAPHSPPMVTFSALSAGENYLRLVCRASGAYPPVDTLTLVSQPQQPEDAPCET
YAGTNADSTGHVGMACVRSDALAGAACAVQHRGVTTSARIVLVPANDGAKVGAYAD
VDADFYADVPPLPEPESDSLAVHALFVAGNTELYVHGTAAGVPSASCRCDTRRCTC
VLAPATWTAGVVRELARAAAHDLLLAVLDVHASGLALNRSSMQVYAECGPAGRRLR
VHNTGTRRQRVCARGACEPAYLVACELLRTDTPAPRRPRMSVQHRRDSSGHYYVCS
AYGFYPKEIVLEMRANRSCDERARLSGFWCRHDPPAPNADGTFFARVFCKAPENAL
LMTCVTRHASRPRALAVPCPRRARTPRERWAALLTVLARVPWSAVLLALAMGAAPL
ACARLVHARSTRTARRARRARRAGKPIPNPLLGLDST

Supplementary Fig 2: Codon optimized mc080 with C-terminal V5 epitope. A) DNA sequence of codon optimised DNA inserted in to RAd-mc080 and B) Translation product showing location of C-terminal tag.

A) DNA Sequence

ATG ACC GGT ACC CTC ATT CTG CTC CTG GCA TGC GTA CTG AAC
GCC ATG GCG CAG TTG CTT GCC CGG GTT TGC ATG GCT GCC GCT
ACA CTC GCA CGC ATG CTC GCC CTT AGC GTG GCG TTT CTG CTG
GCT CTT GCC AGG ACA CGG ACC GGA CTG AGG GCC ATC CTG GTT
GCC CTG CTG CTG CGA GCC TTG CTT AGA GCA CTG CTG GCC CAC
GCA CAT GCG CAC ACA CTG TCC TAC GTA GCTbGCT GTC GTG TAC
ACA CCC GGG AAT GCA CAG CCC TTG CTT CTG GCG GAA GGC TCA
ATC AAC GAC CTG GTG TTC ATG CGG TAT CAC AGA CAA AGC GGC
TCA GTG CTG CCA AGT CCC GAA TGG GCT CCA AGC GTG TAC TTC
CAC GAC GAG CTC TGG ATG CTG AAT GCC CGC GTT GAT GCC CTT
CGG TCC CTG AGT GTG CCT GGT GCA CGA CTG GGG AAT GGC ACC
TTG GGT GCT AGA TCT CTC CAA CTC GCA GTG GGC TGC GAA AAG
GTG GCA GGG GAT GCC AGC TTT TGG GAT CTG GTG TAT GAT GGG
ACT GAG CAG ATC TGT ATG CAC GCT GAT GCC ACC GAG TGT GAG
CCT GGA CTC CCC GTT CAT GCC AGG CTG GCC AAA GAG CGG TGG
ACA AGA CTC GGA GCT CAC AGT CAT GCG CTC GAG CAG CGT TGT
CTG CAG TGG TTG GAA AGG CAT CTG GGA GCA AGG ACC AAC CGA
CCG GTG GTG TCT GTT CCG CTG TTG AGC GTC GTC GCC TAT GCC
GAC GGC TCT GGC ACT CGC CTG CGT TGT ACT GCT TCA GGC TTC
TCT CCC CGA GAT GTG CGG CTG CTG TGG ACT CGC GAT GGA ATT
CCA GGA CCA GAC TAC GAC TTC GTG GAA CCT CGC CCA TCT GGG
GAC GGC AGC TTT CAG CAG TGG GCA GAG CTG GTC GTA GCC GCT
GGC TTG GAG ACA CAC TAT GTC TGC GTA GCC TCC CAT GAC TCC
TGG AAG TCA TCC TGG CGG GCT AGA TGG GAA GAG GGG AAA CGC
AGG GTC GCT ACC AGT GCG AGA GTG GCA CCC TTG GCA ACG ATA
GCC GAG ATG CTT GTC GCC CTG GAA CTC ATG CTG ATT CTG AGG
GAA CGT CGC CTT ACT CTG GGT GCC CTG GCA ACG ATG CTG GCT
TGC AGC ATG CCT AAC CTC CTG CCT CAA GCC CTC AGA GAG AGA
GCT GGA TTT GGT AAG CCA ATC CCT AAC CCG CTC CTA GGT CTT
GAT TCT ACG TGA

MC080 amino acid sequence

MTGTLILLLACVLNAMAQLLARVCMAAATLARMLALSVAFLLALARTRTGLR
AILVALLLRALLRALLAHAHAHTLSYVAAVVYTPGNAQPLLLAEGSINDLVFMRYH
RQSGSVLPSPEWAPSVYFHDELWMLNARVDALRSLSVPGARLGNGTLGARSLQLAV
GCEKVAGDASFWDLVYDGTEQICMHADATECEPGLPVHARLAKERWTRLGAHSHAL
EQRCLQWLERHLGARTNRPVVSVPLLSVVAYADGSGTRLRCTASGFSPRDVRLLWT
RDGIPGPDYDFVEPRPSGDGSFQQWAELVVAAGLETHYVCVASHDSWKSSWRARWE
EGKRRVATSARVAPLATIAEMLVALELMLILRERRLTLGALATMLACSMPNLLPQA
LRERAGFGKPIPNPLLGLDST
